# Predicting Metabolic Syndrome Using Supervised Machine Learning: A Multivariate Parameter Approach †

**DOI:** 10.3390/ijms26209897

**Published:** 2025-10-11

**Authors:** Rodolfo Iván Valdez Vega, Jacqueline Alejandra Noboa-Velástegui, Ana Lilia Fletes-Rayas, Iñaki Álvarez, Martha Eloisa Ramos-Marquez, Sandra Luz Ruíz-Quezada, Nora Magdalena Torres-Carrillo, Rosa Elena Navarro-Hernández

**Affiliations:** 1 Programa de Doctorado en Ciencias Biomédicas, Centro Universitario de Ciencias de la Salud, Universidad de Guadalajara, Guadalajara C.P. 44340, Jalisco, Mexico; rodolfo.valdez2326@alumnos.udg.mx (R.I.V.V.); jacqueline.noboa2713@alumnos.udg.mx (J.A.N.-V.); 2 Departamento de Biología Celular, Fisiología e Inmunología, Institut de Biotecnologia i Biomedicina, Campus de Bellaterra, 08193 Bellaterra, Barcelona, Spain; inaki.alvarez@uab.cat; 3 Departamento de Enfermería Aplicada, Instituto de Investigación en Enfermería y Salud Traslacional, Centro Universitario de Ciencias de la Salud, Universidad de Guadalajara, Guadalajara C.P. 44340, Jalisco, Mexico; lilia.fletes@academicos.udg.mx; 4 Departamento de Biología Molecular, Instituto de Investigación en Enfermedades Crónico-Degenerativas, Centro Universitario de Ciencias de la Salud, Universidad de Guadalajara, Guadalajara C.P. 44340, Jalisco, Mexico; eloisa.ramos@academicos.udg.mx; 5 Departamento de Farmacobiología, Centro Universitario de Ciencias Exactas e Ingenierías, Universidad de Guadalajara, Guadalajara C.P.44430, Jalisco, Mexico; sandra.ruiz@academicos.udg.mx; 6 Departamento de Microbiología y Patología, Centro de Investigación de Enfermedades Infectocontagiosas, Centro Universitario de Ciencias de la Salud, Universidad de Guadalajara, Calle Sierra Mojada No. 950, Colonia Independencia, Guadalajara C.P. 44340, Jalisco, Mexico; 7 Departamento de Biología Molecular, Cuerpo Académico: Inmunometabolismo en Enfermedades Complejas y Envejecimiento (UDG-CA-701), Centro Universitario de Ciencias de la Salud, Universidad de Guadalajara, Calle Sierra Mojada No. 950, Colonia Independencia, Guadalajara C.P. 44340, Jalisco, Mexico

**Keywords:** metabolic syndrome, machine learning, body roundness index, sdLDL-C, high-molecular-weight adiponectin

## Abstract

Metabolic syndrome (MetS) is a complex condition characterized by a group of interconnected metabolic abnormalities. Due to its increasing prevalence, better predictive markers are needed. Therefore, this study aims to develop predictive models for MetS by integrating adipokines, metabolic and cardiovascular risk factors, and anthropometric indices. Data were collected from 381 subjects aged 20 to 59 years (242 women and 139 men) from Guadalajara, Jalisco, Mexico, who were classified as having MetS or non-MetS based on the ATP-III criteria. Four supervised machine learning models were developed—Logistic Regression (LR), Support Vector Machine (SVM), Random Forest (RF), and eXtreme Gradient Boosting (XGBoost)—and their performance was evaluated using the Area under the Curve (AUC), calibration curves, Decision Curve Analysis (DCA), and local interpretability analysis. The RF and XGBoost models achieved the highest AUCs (0.940 and 0.954). The RF and LR models were the best calibrated and showed the highest net benefit in DCA. Key variables included age, anthropometric indices (BRI and DAI), insulin resistance measures (HOMA-IR), lipid profiles (sdLDL-C and LDL-C), and high-molecular-weight adiponectin, used to classify the presence of MetS. The results highlight the usefulness of specific models and the importance of anthropometric variables, cardiovascular risk factors, metabolic profiles, and adiponectin as indicators of MetS.

## 1. Introduction

Metabolic syndrome (MetS) is a common and complex condition affecting many adults worldwide, posing a significant challenge for healthcare systems. It involves a group of related metabolic issues, such as abdominal obesity, insulin resistance, high blood pressure, and abnormal lipid levels. These factors are strongly linked to a higher risk of chronic diseases like type 2 diabetes (T2D) and cardiovascular disease (CVD). Over time, healthcare organizations have created various criteria to define MetS [1,2,3]. Although these definitions often have similar diagnostic standards, there is no universal agreement among clinicians. This lack of standardization explains the variation in MetS prevalence across different populations, which are influenced by factors such as gender, age, race, and environmental and sociocultural elements [4]. Developing a single, unified definition of MetS is crucial for improving the diagnosis, prevention, and management of related chronic conditions.

Machine learning (ML) has become a powerful tool in modern medicine, demonstrating significant potential to enhance risk prediction, early detection, and personalized treatment for various diseases [5,6]. Using large datasets and advanced algorithms, ML models can identify patterns and connections that may not be visible with traditional statistical methods. For MetS, many ML-based approaches have been developed to improve predictive accuracy and facilitate early intervention. These models aim to integrate diverse clinical data to create a more comprehensive framework for risk assessment and management. However, despite promising results from individual studies, there is currently no standardized criterion for MetS across different research, and no universal ML model that fully accounts for all relevant factors in predicting variables such as demographic and physiological factors, dietary influences, biochemical, anthropometric, lipidomic, and proteomic markers, and other aspects related to the onset and progression of MetS. Furthermore, differences in feature selection, data sources, and model performance across studies highlight the need for further research and validation [7,8,9,10,11,12,13,14].

To improve the prediction of MetS, this study aims to develop machine learning models: Logistic Regression (LR), Support Vector Machine (SVM), Random Forest (RF), and eXtreme Gradient Boosting (XGBoost) by combining adipokines, metabolic and cardiovascular risk factors, and various anthropometric indices, offering a more comprehensive tool for early detection and risk assessment of MetS.

## 2. Results

### 2.1. Demographic, Clinical, and Anthropometric Variables of the Study Subjects

Table 1 displays the demographic, metabolic, anthropometric, and biochemical characteristics of the study participants, grouped by MetS status. The prevalence of MetS was 22.04% (n = 84), with women mainly represented in both groups. Compared to the non-MetS group, individuals with MetS had significantly higher levels of systolic and diastolic blood pressure, triglycerides, total cholesterol, LDL-C, sdLDL-C, fasting glucose, insulin, and HOMA-IR (*p* < 0.001). They also had lower HDL-C and QUICKI values (*p* < 0.001). Anthropometric measures, including BMI, WC, BRI, and DAI, were also notably higher (*p* < 0.001), indicating increased central adiposity. Furthermore, total adiponectin (AdipoQT) and high-molecular-weight adiponectin (AdipoQHMW) levels were significantly lower in the MetS group (*p* < 0.001), while no significant differences were observed in chemerin, irisin, CRP, or C3.

### 2.2. Data Pre-Processing

To develop the machine learning models, we selected anthropometric variables (ABSI, AVI, BAI, BFI, BFR, BMI, BRI, CI, TAA, VAI), metabolic and lipid profile variables (Apo A, Apo B, Insulin, LDL-C, sdLDL-C), adipokines, and inflammatory markers (AdipoQT, AdipoQHMW, chemerin, irisin, CPR, C3), metabolic indices (DAI, HOMA-IR), age, and sex. The selection was based on significant correlations with MetS parameters, excluding variables with high collinearity (rho > 0.9), and differences between groups.

**Figure 1:**
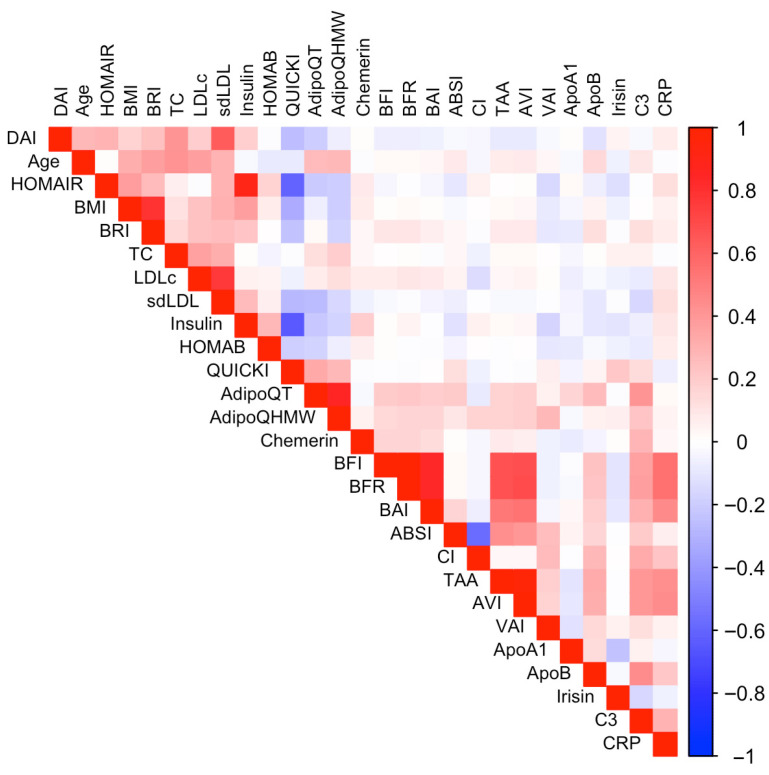
Correlation matrix of predictor variables considered for model selection. The heatmap displays the Pearson correlation coefficients among variables considered as potential predictors. Positive correlations are shown in red, and negative correlations in blue, with more intense colors indicating a stronger relationship.

Furthermore, to minimize redundancy and prevent overfitting in the predictive model, we conducted a correlation analysis on all candidate variables. Pearson correlation coefficients were calculated and displayed as a heat map, where red and blue gradients indicate positive and negative correlations, respectively (Figure 1). Variables with a correlation coefficient above 0.85 (AVI, TAA, BFR, BMI, and insulin) were excluded from further analysis to ensure a more independent set of predictor variables and enhance the model’s generalizability.

The correlation matrix illustrates the core features of MetS, showing strong positive associations between anthropometric indices, insulin resistance (HOMA-IR and insulin), sdLDL-C, and inflammatory markers (CRP and C3). In contrast, adiponectin displayed inverse correlations with these parameters, highlighting its protective role. Together, these patterns reflect the interplay of obesity, dyslipidemia, and low-grade inflammation as central mechanisms driving MetS and its cardiometabolic risk.

Missing data were handled depending on the missing values for each variable and the type of data. Variables with less than 5% missing data were imputed using the median, while those with a higher percentage of missing data (10–20%), such as adiponectin (AdipoQT, AdipoQHMW), were imputed using k-nearest neighbors (KNN, k = 5) to preserve multivariate relationships.

#### 2.2.1. Recursive Feature Elimination with Cross-Validation

To perform this analysis, feature selection was carried out using recursive feature elimination (RFE) within a cross-validation process for each algorithm. Table 2 presents a summary of the resampling results for each model, including AUC-ROC and accuracy metrics for the model with all predictors compared to those obtained using the RFE process.

**Figure 2:**
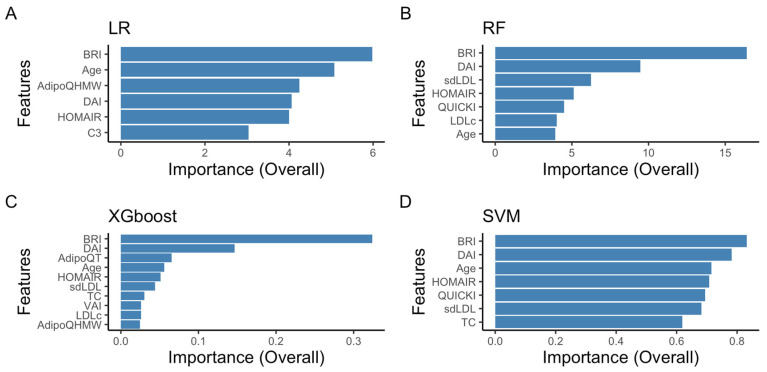
Ranking of feature importance from recursive feature elimination with cross-validation using four classifier models. The blue bars show features selected by the recursive feature elimination with cross-validation algorithm, with the importance (Overall) of each bar indicating the feature’s rank. Panel (**A**) shows the feature importance results for the LR model, panel (**B**) for the RF model, panel (**C**) for the XGBoost model, and panel (**D**) for the SVM model.

All models achieved a significant reduction in important variables. The RF model showed the highest accuracy (0.875) with a reduced dataset of seven selected variables, followed by the XGBoost model (0.854), which used ten variables. Figure 2 displays the variable importance according to RFE for each model. Feature importance primarily includes anthropometric variables, with the BRI and DAI leading the list, encompassing a diverse group of variables. Among these, sdLDL-C, LDL-C, HOMA-IR, QUICKI, AdipoQHMW, and age stand out.

#### 2.2.2. Cross-Validation: Evaluating Model Performance

Once the recursive elimination process is complete, the selected feature set undergoes 10-fold cross-validation (10 × 10 repeated CV) within the training set. The evaluation metrics for the trained models are displayed in Table 3. We employed the Friedman test to compare model performance based on three metrics: the area under the receiver operating characteristic curve (AUC-ROC), sensitivity, and specificity. In all cases, we observed statistically significant differences between models (*p* < 0.05), indicating that at least one model performed differently from the others. Subsequently, post hoc tests (adjusted Bonferroni test) were performed to identify specific differences between each pair of models.

As shown in Table 3, the LR model achieved the highest AUC-ROC [0.910 (±0.048)], which was significantly higher (*p* < 0.001) than the XGBoost model [0.867 (±0.063)]. Additionally, the LR model demonstrated high sensitivity values [0.816 (±0.069)] and was statistically better than the RF [0.781 (±0.088)] and XGBoost models [0.759 (±0.090)]. Similarly, the LR model recorded the highest specificity [0.859 (±0.146)], and this difference was statistically significant compared to the XGBoost model [0.778 (±0.179)]. Overall, the LR model provided the best balance of sensitivity and specificity, resulting in superior overall performance. The optimal hyperparameters for each model are listed in Appendix A.

#### 2.2.3. Test Data Performance

Table 4 displays the evaluation results of the four supervised classification models tested on the validation set. As shown, all four models achieved accuracy rates from 0.80 (95% CI, 0.67–0.90) for XGBoost to 0.88 (95% CI, 0.76–0.96) for RF, exceeding the missing information rate (*p* < 0.01), indicating that the findings are not due to chance. Among them, the RF model had the highest balanced accuracy at 0.88 (95% CI, 0.77–0.96). Similarly, the detection of MetS cases was satisfactory across all models, with sensitivity values ranging from 0.70 (95% CI, 0.46–0.88) for XGBoost to 0.85 (95% CI, 0.62–0.97) for LR and RF. The RF model demonstrated the highest sensitivity at 0.85 (95% CI, 0.62–0.97). The McNemar test showed no significant difference between false positives and false negatives in any model (*p* > 0.05), suggesting no systematic bias in the predictions.

**Figure 3:**
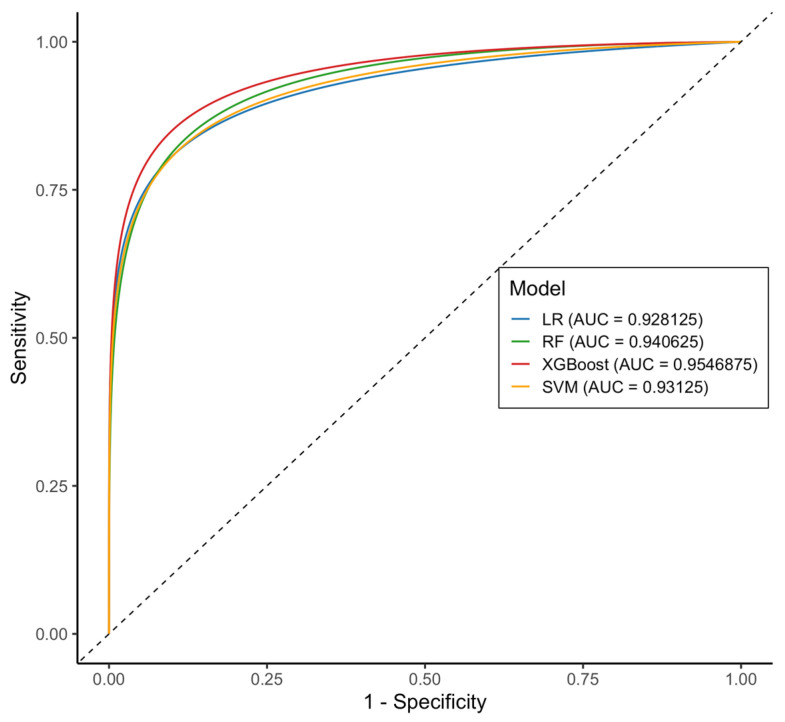
Comparative analysis of AUC-ROC between the four models. According to the AUC-ROC, the XGBoost model was the best model (0.954, red), followed by RF (0.940, green), SVM (0.931, yellow), and LR (0.928, blue). Abbreviations: AUC-ROC, area under the receiver operating characteristic curve.

Furthermore, the AUC-ROC values demonstrated strong discriminatory ability across all models. XGBoost and RF models achieved the highest AUC-ROC scores (0.954 [95% CI: 0.87–1.00] and 0.94 [95% CI: 0.88–1.00], respectively), followed by SVM (0.931 [95% CI: 0.86–0.99]) and LR (0.93 [95% CI: 0.86–0.99]) models. DeLong’s test revealed no statistically significant differences between the models (*p* > 0.05), suggesting comparable discriminative performance. These findings indicate that all four classifiers demonstrated excellent predictive accuracy for identifying individuals with MetS (Figure 3).

**Figure 4:**
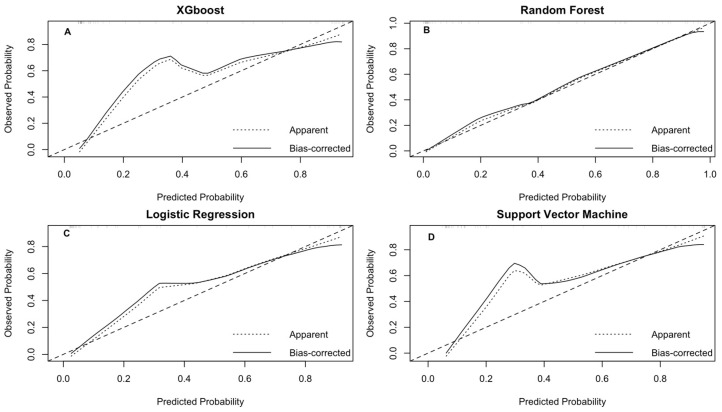
Calibration curves of the four predictive models for MetS in validation data. The curves show the relationship between predicted probabilities and observed rates of MetS in the validation set. The dotted diagonal line indicates perfect calibration, where predictions exactly match observed values; the solid line represents the predictions adjusted through bootstrap. Panel (**A**) shows the calibration curve for the XGBoost model, panel (**B**) for the RF model, panel (**C**) for the LR model, and panel (**D**) for the SVM model.

After assessing the discriminatory ability of each model, calibration plots were generated to demonstrate the reliability of the predicted probabilities (Figure 4). The LR and RF models closely followed the ideal line, with a brief score of 0.08 and 0.10, respectively, indicating well-calibrated risk estimates. Moreover, the calibration curve analysis showed that for the RF model, the intercept was −0.068 and the slope was 0.907, and for the LR model, the intercept was 0.05 and the slope was 0.96, indicating minimal overfitting and good agreement between predicted and observed probabilities. In contrast, although the SVM and XGBoost models exhibited high AUC-ROC values, they showed notable deviations. The SVM model overestimated the lower range (0.2–0.4) and underestimated the upper predicted probabilities (>0.6).

**Figure 5:**
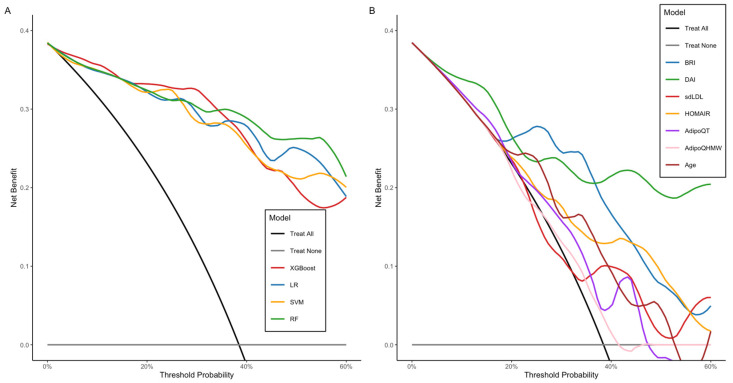
Decision curve analysis comparing four models and individual predictors. The *x*-axis shows the threshold probability (0–60%), while the *y*-axis indicates Net Benefit. The gray lines (Treat None) represent the expected net benefit without any treatment or intervention, showing that this strategy does not provide benefits (net benefit > 0). The black line (Treat ALL) indicates treatment or intervention given to all patients. The colored lines correspond to each evaluated model (panel **A**) and individual predictors (panel **B**).

Furthermore, to assess the predictive models, we conducted a decision curve analysis (DCA). It began with the diagnostic threshold probability for MetS and the relative significance of false-positive and false-negative results. The net benefit of each model was calculated at various probabilities, and by plotting the net benefit against the threshold probability, the decision curve was generated. Figure 5, panel A displays the decision curves for the four prediction models within the range of interest (0–60%). The net benefit summary shows that all four models outperformed the strategy of not treating any or all patients, confirming their added value in predicting MetS. The RF model had the highest average benefit (NB = 0.294), followed by the LR (NB = 0.281), XGBoost (NB = 0.274), and SVM (NB = 0.273) models. At lower thresholds (10–20%), all four models demonstrate similar net benefits. At these levels, the “treat everyone” strategy does not significantly penalize false positives. As the threshold rises (20–50%), the RF and LR models maintain more consistent net benefits, while the SVM and XGBoost models display more noticeable fluctuations, indicating greater sensitivity to threshold selection.

On the other hand, analysis by individual predictors (Figure 5, panel B) revealed a significant overall clinical benefit. DAI led the list with the highest average benefit (mean NB = 0.236), followed by BRI (mean NB = 0.188) and HOMA-IR (mean NB = 0.159). Other predictors, such as age, sdLDL-C, AdipoQT, and AdipoQHMW, showed smaller average net benefits (mean NB ≤ 0.14), approaching the “treat all” curve, indicating lower utility when used alone. DAI remained above the 40–50% threshold, demonstrating its clinical usefulness across a wide range of patients. BRI and HOMA-IR showed positive benefits of approximately 30–40% of the threshold, but with minor effects. Variables such as age, sdLDL-C, AdipoQT, and AdipoQHMW dropped sharply toward “do not treat” values when the threshold exceeded 20–25%, showing a reduced ability to maintain clinical utility on their own.

#### 2.2.4. Random Forest and Logistic Regression as the Optimal Models: Interpretability Analysis

**Figure 6:**
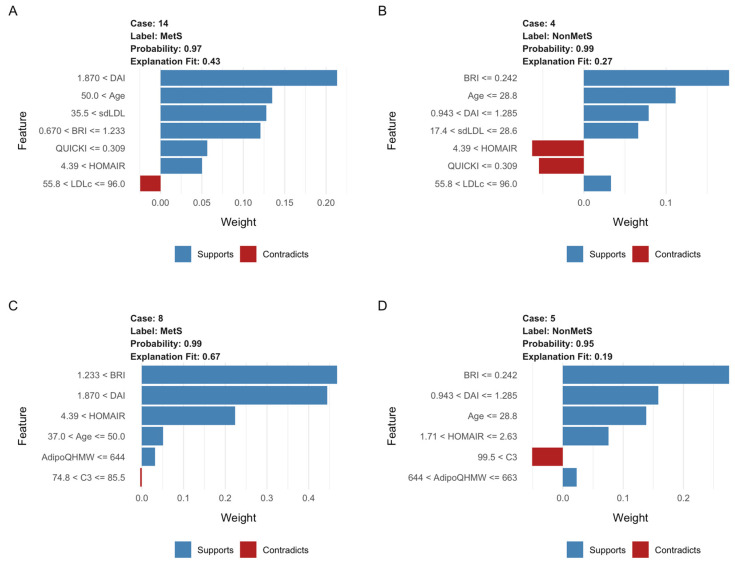
LIME analysis results for specific cases using RF and LR. Four representative cases of LIME analysis (MetS and non-MetS) were selected for the two best models. LIME plots illustrate the key variables that affect the classification of MetS. Blue bars indicate variables that positively influence the prediction of MetS, while red bars show variables that oppose it. Panels (**A**,**B**) correspond to the RF model for classifying MetS and non-MetS, respectively (Case 14, Probability: 0.97; Case 4, Probability: 0.99). Panels (**C**,**D**) show the LR model for classifying MetS and non-MetS, respectively (Case 8, Probability: 0.99; Case 5, Probability: 0.95). The length of each bar reflects the relative importance of that variable in the final classification.

**Figure A1:**
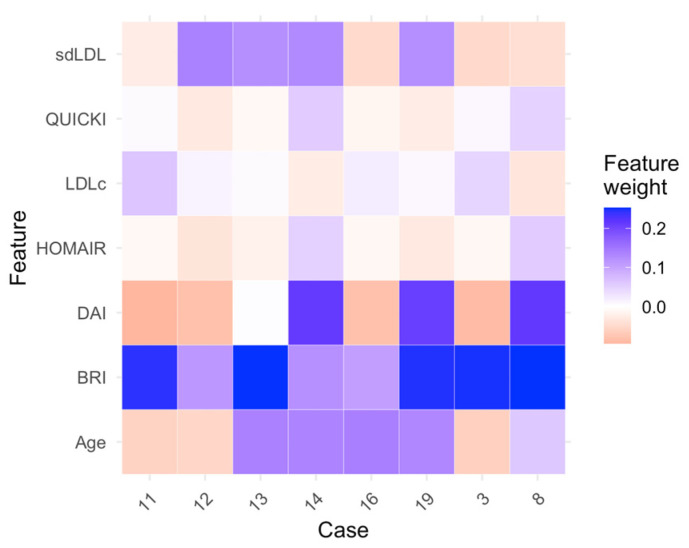
Average feature weight of variables in MetS prediction according to LIME in the RF model. The figure displays a heatmap showing the average contribution of each variable in predicting MetS with the evaluated model. The colors indicate the average influence of each feature on the classification: blue signifies a positive contribution, while red signifies a negative one. The brightness of the color reflects the strength of each variable’s relative influence.

Among the models evaluated, the RF and LR classifiers were selected as the best options due to their superior performance on several key metrics, and they proved to be the most well-calibrated models. The analysis of local explanations using LIME helped identify the variables with the most significant influence on model predictions. In the case of RF (Figure A1, Appendix B), variables such as BRI, sdLDL-C, and age emerged as key factors in classifying MetS. At the same time, DAI, HOMA-IR, and QUICKI acted as conflicting variables to MetS and assisted in identifying negative cases. For the RF example (Figure 6A,B panels), in a positive MetS diagnosis (case 14, probability = 0.97), variables such as DAI (<1.87), age (<50), sdLDL-C (<35.5), and BRI strongly supported the classification. At the same time, low QUICKI (0.309) and intermediate LDL-C levels worked against it. Conversely, in a negative MetS diagnosis (Case 5, probability = 0.99), low BRI values (0.242), age (28.8 years), and sdLDL-C (17.4–26.8) favored the non-MetS prediction, although a high HOMA-IR (4.39) partially contradicted this.

**Figure A2:**
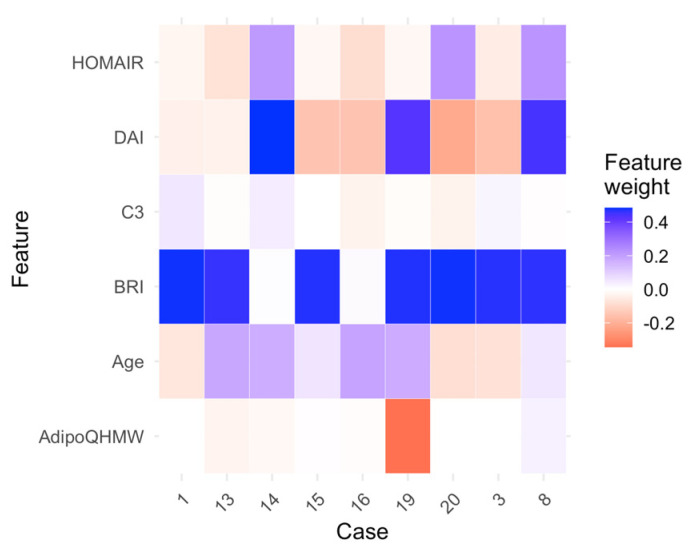
Average feature weight of variables in MetS prediction according to LIME in the LR model. The figure displays a heatmap showing the average contribution of each variable in predicting MetS with the evaluated model. The colors indicate the average influence of each feature on the classification: blue signifies a positive contribution, while red signifies a negative one. The brightness of the color reflects the strength of each variable’s relative influence.

In the LR model (Figure A2, Appendix C), variables such as BRI, DAI, and age were identified as the main predictors for distinguishing between positive and negative cases. At the same time, HOMA-IR, AdipoQT, and AdipoQHMW acted as contradictory variables to the MetS classification. Moreover, in the LR example (Figure 6C,D panels), a positive MetS diagnosis (case 8, probability = 0.99) was associated with high values of DAI (<1.87), BRI (<1.233), and HOMA-IR (<4.39). In contrast, negative cases (case 5, probability = 0.95) were characterized by high BRI values (<0.242), age (28.6 years), and HOMA-IR (1.71–2.63), along with typical ranges of high-molecular-weight adiponectin (644–663).

## 3. Discussion

Previous studies have shown that MetS is a significant risk factor for conditions such as type II diabetes and cardiovascular diseases (CVDs). Therefore, with its increasing prevalence, more precise predictive markers are needed. In this study, we include a broader range of biomarkers and develop a predictive model for MetS that combines demographic data, clinical biomarkers, and anthropometric measurements, using four supervised machine learning classification algorithms. The resulting model can aid in the early detection of MetS and support more informed clinical decision-making. Additionally, our predictive model demonstrates potential in accurately identifying true-positive cases with significant risk factors early, enabling more efficient use of medical resources in clinical practice.

We observed a 22% prevalence of MetS in our study population, which closely aligns with the findings reported by Hossain et al. in a Korean population [15]. Similarly, the significant increases in anthropometric and clinical parameters among the MetS group are consistent with results from other studies [16]. These notable differences in most variables between the MetS and non-MetS groups helped us select specific factors for developing prediction models for MetS and identifying the most effective one. We employed supervised classification algorithms: LR, RF, XGBoost, and SVM. Each model showed promising accuracy, ranging from 80.7% to 86.5%. Their ability to identify MetS cases was also satisfactory, with sensitivity values ranging from 70% to 90%. However, the models based on XGBoost and RF performed the best according to the area under the curve, scoring 0.946 and 0.940, respectively. The RF and LR models demonstrated the best calibration and the highest net benefit in the DCA.

It has already been reported that complex learning models, such as RF, XGBoost, SVM, and neural networks, outperform traditional statistical models, like LR, in classifying MetS [15,17,18,19,20]. This shows that imbalance ratios affect the performance of each model in sampling methods and the average fold change. A previous study using blood biomarker analysis evaluated an SVM-based cascade classifier for the automated diagnosis of MetS. The researchers achieved an average accuracy of about 84% in correctly classifying BMI and 74% in accurately classifying systolic blood pressure. Overall, the system demonstrated 84% accuracy in predicting MetS [21]. After analyzing the four models, key variables included age, anthropometric indices (BRI and DAI), insulin resistance measures (HOMA-IR), lipid profiles (sdLDL-C and LDL-C), and high-molecular-weight adiponectin were identified as better predictors of MetS.

The BRI index was developed over ten years ago as a comprehensive tool that utilizes height, waist, and hip circumference to estimate total body fat percentage and visceral adipose tissue. It has also been utilized as a visual tool for assessing overall health status [22]. Recent studies have demonstrated that the BRI index is associated with all-cause mortality, underscoring its potential beyond merely estimating adiposity [23,24]. Therefore, all this evidence strongly supports our predictive models, endorsing the inclusion of the BRI index as a key parameter for predicting MetS. Similarly, a previous meta-analysis found that the pooled estimated AUC for the BRI index was larger than those for BMI, waist-to-hip ratio (WHR), ABSI, and BAI, such as waist circumference, and smaller than the waist-to-height ratio (WHtR). However, the differences between the BRI and BMI, waist circumference, and WHtR in predicting MetS were not statistically significant [25].

Additionally, the DAI index, a key measure of adipose dysfunction [26], has been included in predictive models. A previous study demonstrated that the DAI index had a strong ability to distinguish MetS (AUC = 0.921) compared to other anthropometric measures [27]. Another study involving women with Polycystic Ovary Syndrome found that the DAI index had an AUC of 0.86, with a sensitivity of 74% and a specificity of 80% for diagnosing MetS [28]. Similarly, variables such as HOMA-IR, sex, age, and diabetes mellitus status have been incorporated into predictive models, and their accuracy was evaluated using multiple logistic regression to predict the onset of MetS. The model’s performance showed an AUC of 0.819, with a sensitivity of 80% and a specificity of 68.9% [29].

Although sdLDL-C has been recognized for over twenty years as an important biomarker of cardiovascular disease, its direct connection with MetS has not yet been conclusively proven [30]. However, we observed that the group with MetS had higher sdLDL-C levels, supporting earlier research that demonstrates a link between sdLDL-C and MetS [31,32]. Therefore, our findings suggest that sdLDL-C could serve not only as a marker for cardiovascular disease but also as a strong predictor of MetS. Another parameter our model identified as a key predictor of MetS was AdipoQT. Discovered more than twenty years ago, it exhibits antidiabetic, antiatherosclerotic, and anti-inflammatory properties. Its levels decrease with increasing visceral fat [33], and its concentration varies based on sex, age, and ethnicity [34]. It has been reported that a low adiponectin/leptin ratio is associated with increased levels of inflammatory markers in adipose tissue, leading to dysfunction. This suggests that a low adiponectin/leptin ratio may be a characteristic feature of obesity and MetS [35,36,37].

Our results emphasize the importance of a comprehensive analysis that includes anthropometric variables, cardiovascular risk factors, metabolic profiles, and adiponectin as predictive indicators of MetS. Unlike the studies previously discussed, which analyzed these variables separately or alongside other factors, our approach demonstrates their predictive value when considered together.

Additionally, during the interpretability analysis of the LR and RF models using LIME, we found that, in most cases examined individually, variables such as BRI, DAI, age, adiponectin, sdLDL-C, and HOMA-IR played a significant role in predicting MetS. This aligns with a phenotype of dysfunctional adiposity and metabolic disruption, enhancing the clinical utility for diagnosing MetS. Several studies have investigated these markers as potential risk predictors [38,39,40,41,42]. LIME’s analysis across different cases showed how the most essential variables influence the prediction of MetS. When the BRI index exceeds 5.5, DAI is higher than 3.0, and HOMA-IR ranges between 1.71 and 4.39, the likelihood of predicting MetS increases. Age also plays a role; being over 45 years old increases the likelihood of MetS prediction. A previous study found that the HOMA-IR threshold decreases from 3.46 to 2.05 when examining the components of MetS. Additionally, a value of 1.85 in men was identified as a predictor of MetS risk [43]. For BRI predicting MetS risk, a cutoff point of 6.2 was previously used for women in Asian populations, while in Latin America, the optimal cutoff was 4.0 for women [44].

There are other tools, such as the Framingham Risk Score (FRS) and the modified Finnish Diabetes Risk Score (FINDRISC), which are helpful in the clinical setting for predicting MetS [45]. The FRS is elevated in people with MetS [46], and FINDRISC has good discrimination (AUC-ROC: 80.9%) with a sensitivity of 74.0% and a specificity of 75.5% for predicting MetS [45]. The development of our models also included variables related to cardiovascular and diabetes risk that are considered in these tools; however, our main goal was to create a more robust model that incorporates new anthropometric indices, metabolic profiles, and the role of adiponectin as an indicator of its impact on the development of metabolic syndrome.

We must interpret these results carefully because of several limitations. First, although the study population provided valuable insights, its size limits the extent to which the findings can be applied broadly. Additionally, the data were collected from a population in a single region of western Mexico, which may not fully represent the diversity of other geographic areas or ethnic groups, further limiting generalizability. Lastly, our study did not include validation with an external cohort. Moreover, this study lacked historical data from earlier years of each participant, which could have offered a broader view of trends over time. Similarly, the absence of detailed nutritional information from participants may have limited the ability to assess the impact of diet on the studied parameters. To address current limitations related to sample size and geographic representation, future research will involve collaborations with regional hospitals to expand the dataset and improve population diversity, as well as the development of a multicenter cohort for external validation. This will help enhance the robustness and predictive accuracy of our models.

Despite all the points mentioned above, our study has several strengths. We evaluate a wider range of anthropometric indices, expanding beyond traditional measures to provide a more comprehensive view of body composition. Additionally, we incorporate biochemical profiles and serum adipokine levels to support the use of machine learning algorithms aimed at improving MetS prediction. Although machine learning is a relatively new tool in this field, it has gained popularity among many research groups. Continued application of this approach will enhance our understanding and lead to more accurate predictive models in future studies.

## 4. Materials and Methods

### 4.1. Study Population

Demographic, clinical, and anthropometric data were collected from 381 subjects aged 20 to 59 years (242 women and 139 men) from the urban population of Guadalajara, Mexico, who attended the Instituto de Investigación en Enfermería y Salud Traslacional del Departamento de Enfermería Aplicada del Centro Universitario de Ciencias de la Salud de la Universidad de Guadalajara, Guadalajara, Jalisco, México. All participants provided written informed consent before being included, and the protocol was approved by the Ethics and Research Committee of the Antiguo Hospital Civil de Guadalajara “Fray Antonio Alcalde,” O.P.D. HCG/CEI-0835/22, No. 130/22. The data collected comes from a cohort of more than 600 participants from 2015 to 2024 [47,48,49].

### 4.2. Blood Sample Collection

Peripheral blood samples were collected in the morning after a 12 h fast from all participants. Both serum and plasma were separated using standard laboratory procedures, aliquoted, and stored at −20 °C until analysis.

### 4.3. Biochemical Parameters

Each participant was evaluated for the following biochemical parameters: fasting glucose (mg/dL) (RANDOX Laboratories Limited, Crumlin, UK), total cholesterol (mg/dL), triglycerides (mg/dL), HDL-C (mg/dL), LDL-C (mg/dL), and sdLDL-C (RANDOX Laboratories Limited, Crumlin, UK) using the immunoturbidimetric method, according to the manufacturer’s instructions. All assays were conducted under the quality control procedures provided by the corresponding commercial kits, ensuring compliance with the recommended standards of precision and reproducibility. Additionally, insulin (μIU/mL) (Human Immunoassay ALPCO Diagnostics, Salem, NH, USA), total adiponectin (AdipoQT), high-molecular-weight adiponectin (AdipoQHMW) (HWM and Total Adiponectin ELISA; Cat. No. 47-ADPHU-E01; ALPCO Keewaydin Drive, Salem, NH, USA), and chemerin (Cat No. MBS029209, MyBioSource, San Diego, CA, USA) were measured with an immunoassay ELISA, following the manufacturer’s instructions.

### 4.4. Blood Pressure Measurements

A nurse measured systolic and diastolic blood pressure using a sphygmomanometer. To do this, each participant was asked to sit in a chair with their feet flat on the floor and their back straight, resting their arm on a surface at heart level. The sphygmomanometer is placed around the arm and inflated to compress the artery. As the cuff deflates, the sounds of blood flow are listened to with a stethoscope to record the systolic pressure (the higher number, when the heart beats) and the diastolic pressure (the lower number, between beats).

### 4.5. Metabolic and Anthropometric Parameters

Anthropometric data included height (in cm), weight (in kg), and waist and hip circumferences (in cm), as well as the calculated body mass index (BMI) in kg/m^2^. Body composition parameters were assessed using electrical bioimpedance. Body adiposity status was evaluated and calculated using the following indices and formulas: ABSI (a shape index): waist circumference/(BMI^2/3^ * Height^1/2^) [50]; AVI (abdominal volume index): (2 * waist circumference^^2^ + 0.7 * (waist circumference − hip circumference)^^2^)/1.000 [51]; BAI (body adiposity index): (hip circumference/height^^1.5^) − 18 [46]; BFI (body fat index): total body fat/height; BFR (body fat ratio): body fat mass/total body mass * 100; BRI (body roundness index): 364.2 − 365.5 * √(1 − (WC/2π)^^2^/(0.5 * height)^^2^) [24]; CI (conicity index): waist circumference/(0.109 * (weight/height)^^0.5^) [52]; DAI (dysfunctional adipose index), DAI_men_: waist circumference/(22.79 + (2.68 * BMI)) * (triglycerides/1.37) * (1.19/HDL-C) for men, and DAI_women_: (waist circumference/(24.02 + (2.37 * BMI))) * (triglycerides/1.32) * (1.43/HDL-C) for women [53]; TAA (tissue adiposity area): total body fat; and VAI (visceral adiposity index), VAI_men_: (waist circumference)/((39.68 + 1.88 * BMI) * (TG/1.03) * (1.31/HDL-C)), VAI_women_: (waist circumference)/((39.58 + 1.89 * BMI) * (triglycerides/0.81) * (1.52/HDL-C)) [54], where waist circumference, hip circumference, and height are in cm, weight in kg, triglycerides and HDL-C in mmol/L.

### 4.6. Definition of Metabolic Syndrome

To identify subjects with MetS, we used the criteria defined by the National Cholesterol Education Program’s (NCEP) Adult Treatment Panel III (ATP III). This definition is one of the most widely used standards for MetS. It includes the key features of hyperglycemia/insulin resistance, visceral obesity, atherogenic dyslipidemia, and hypertension. Additionally, it relies on measurements and laboratory results that are easily accessible to physicians, supporting both clinical and epidemiological applications. According to the ATP III definition, MetS is present if three or more of the following five criteria are met: waist circumference > 102 cm (men) or >88 cm (women), blood pressure ≥ 130/85 mmHg, fasting triglycerides (TG) level over 150 mg/dL, fasting high-density lipoprotein (HDL) cholesterol levels < 40 mg/dL (men) or <50 mg/dL (women), and fasting blood glucose ≥100 mg/dL [55,56,57].

### 4.7. Development of Predictive Models Using Supervised Machine Learning

#### 4.7.1. Data Selection and Preprocessing

The dataset from patients included 381 data points. The key predictors of MetS were identified based on their clinical importance and descriptive analyses. To prevent overfitting, variables that are part of the MetS diagnosis according to the ATP-III criteria were excluded. To address potential class imbalances in the training set, an up-sampling technique was used for random resampling. For data preprocessing, the R package “caret” (version 7.0-1) was used, which offers various functions to prepare predictor data. These functions include removing predictors with zero or near-zero variance, handling correlated predictors, centering and scaling, and performing median and Knn imputation [58]. Given the relatively small sample size, the best strategy for data splitting is to include all the subjects in the training set to maximize the information used for estimating parameters and selecting predictors. However, there is a risk of selection bias. To avoid this bias, a simple time-division strategy was implemented, which involves dividing the data into training (80%) and test (20%) sets on an annual basis. The last two years of the study period were used as test data [59,60].

#### 4.7.2. Cross-Validation and Feature Selection

The open-source R package “caret” was used to develop and evaluate four supervised machine learning (ML) algorithms aimed at identifying variables associated with MetS: Logistic Regression (LR), Support Vector Machine (SVM), Random Forest (RF), and eXtreme Gradient Boosting (XGBoost) [61,62]. Feature selection was performed using recursive feature elimination (RFE) for each algorithm. This method identifies the most critical subset of predictors that optimizes model performance. RFE was carried out using 10-fold cross-validation repeated 10 times (10 × 10 repeatCV) on the training data to ensure stability and reduce overfitting. During each RFE step, internal resampling was used to calculate evaluation metrics, including AUC-ROC, accuracy, sensitivity, and specificity, which guided the choice of the best feature set [63]. After recursive elimination, the selected feature set was validated with 10-fold cross-validation (10 × 10 repeatCV) within the training set to ensure robustness and prevent overfitting. For each algorithm, hyperparameter tuning was performed using a grid search with the caret’s tuneGrid function. The models’ performance, assessed via cross-validation, was evaluated using accuracy, AUC-ROC, specificity, and sensitivity [59].

#### 4.7.3. Model Performance Evaluation

The model’s performance was then evaluated on the test dataset. Predicted class labels were generated as probabilities for the MetS group. These predictions were compared with actual results using confusion matrices, which provided metrics such as sensitivity, specificity, and accuracy. Additionally, the AUC-ROC values of the curves were calculated using the predicted probabilities to assess the discriminatory power of each model. The AUC serves as a measure of accuracy (A) and is a comprehensive metric for evaluating the performance of the ROC curve. A higher AUC-ROC value indicates better predictive performance of the model [64,65]. To generate the calibration curves for all models, the “rms” package (R package version 8.0-0) was used. Validation was performed using bootstrap (B = 200 replicates) to reliably estimate the calibration curve [66,67].

Decision curve analysis (DCA) was also employed to examine the relationship between benefit and risk at various cutoff points across different models. It helps identify the threshold probability range where a model performs best, evaluates the benefits, and supports choosing the most suitable model among several options. The DCA calculates the ‘net clinical benefit’ for one or more predictive models across a range of threshold probabilities, considering the relative harms of false positives and false negatives. The analysis was performed using the “d. curves” package (Decision Curve Analysis for Model Evaluation, R package version 0.5.0) [68,69,70]. To interpret the best model, a Local Surrogate Model was built using the Local Interpretable Model-agnostic Explanations (LIME) method. This technique helps clarify individual predictions made by the model [71].

#### 4.7.4. Statistics Analysis

The study population was described using mean and standard deviation (SD) or median and interquartile range (IQR) for continuous variables, as well as frequency (n) and proportion (%) for categorical variables. Student’s *t*-test or U-Mann–Whitney test was used for continuous variables, and the Chi-squared test was used for categorical variables (JASP program v0.18.1, RStudio v2023.12.1 + 402 with R v4.2.1). For model evaluation, a McNemar test was performed to compare sensitivity and specificity within and between models, and the DeLong test was applied to assess statistical differences between the AUCs of the models. The Friedman and post hoc tests were utilized to evaluate multiple models and observe their differences. A *p* value of <0.05 was considered statistically significant [64].

## 5. Conclusions

To identify additional variables that contribute to defining MetS, this study evaluated four machine learning models. The RF and LR models performed the best, surpassing the others in calibration and yielding the highest net utility in the ACD. Additionally, using interpretability tools such as LIME, we examined how variables influence interpretation and decision-making in each model. Consequently, the LR and RF models identified key anthropometric variables, such as BRI, DAI, HOMA-IR, QUICKI, sdLDL-C, high-molecular-weight adiponectin, and age, as the most critical factors in diagnosing MetS. Overall, these findings highlight the potential of machine learning in diagnosing MetS and provide a strong foundation for future clinical research focused on personalized treatment and early prevention. However, it is essential to note that the relevance and impact of these variables may vary depending on the population studied; therefore, external validation in different clinical and demographic settings is necessary.

## Figures and Tables

**Table 1:** Demographic, metabolic, and anthropometric parameters of the study subjects.

Groups	MetS	Non-MetS	*p* Value
n	84	297	
Sex (Men/Women)	29:55	110:187	
Age	44 ± 14	35 ± 19	
Blood Pressure (mmHg)			
Systolic	124 (13)	108 (15)	<0.001
Diastolic	81 (14)	71 (15)	<0.001
Lipid Profile			
Apo A	129.5 (31.75)	135.0 (50.25)	0.207
Apo B	104 (52)	88.5 (50.25)	0.089
HDL-C (mg/dL)	38.6 (8.6)	43.0 (13.3)	<0.001
LDL-C (mg/dL)	110.9 (68.7)	101.4 (54.5)	0.043
sdLDL-C (mg/dL)	44.1 (27.9)	23.1 (27.82)	<0.001
Total cholesterol (mg/dL)	211 (57)	192 (53)	<0.001
Triglycerides (mg/dL)	252 (134)	119 (64)	<0.001
Insulin Resistance Status			
Fasting glucose (mg/dL)	101.0 (22.5)	86.0 (22.0)	<0.001
Fasting insulin (μUI/mL)	20.6 (17.5)	12.8 (11.7)	<0.001
HOMA-IR	5.1 (3.9)	2.5 (2.4)	<0.001
HOMA-B	15.2 (15.4)	15.7 (14.6)	0.008
QUICKI	0.31 (0.04)	0.35 (0.06)	<0.001
Body Adiposity Status Evaluation			
ABSI	0.080 (0.008)	0.80 (0.009)	0.596
AVI	17.58 (8.53)	16.07 (17.72)	0.531
BAI	31.25 (6.94)	31.36 (9.32)	0.666
BFI	8.88 (5.15)	8.29 (5.84)	0.334
BFR	8.88 (5.15)	8.29 (5.73)	0.302
BMI (kg/m^2^)	33.3 (6.9)	26.7 (6.1)	<0.001
BRI	1.5 (0.9)	0.5 (0.6)	<0.001
CI	1.25 (0.163)	1.23 (0.168)	0.518
DAI	2.7 (1.8)	1.3 (0.9)	<0.001
TAA	688.00 (341)	636.50 (284.5)	0.533
VAI	0.20 (0.200)	0.20 (0.198)	0.315
WC	107.3 (15.5)	82.8 (18.9)	<0.001
Adipokines and inflammation markers			
AdipoQT (ng/mL)	4252 (4125)	7108 (4728)	<0.001
AdipoQHMW (ng/mL)	981 (1199)	2582 (2991)	<0.001
Chemerin (ng/mL)	129 (66)	147 (89)	0.191
Irisin	4560 (1994)	4889 (2124)	0.380
CRP	6.45 (5.40)	5.48 (4.32)	0.058
C3	108.5 (47.25)	102.0 (42)	0.232

Mann–Whitney *U* test, IQR value. Abbreviations: HDL-C, high-density lipoprotein cholesterol; LDL-C, low-density lipoprotein cholesterol; sdLDL-C, small low-density lipoprotein cholesterol; HOMA-IR, homeostasis model assessment-estimated insulin resistance; HOMA-B, homeostasis model assessment of β-cell function; QUICKI, quantitative insulin-sensitivity check index; ABSI, a shape index; AVI, abdominal volume index; BAI, body adiposity index; BFI, body fat index; BFR, body fat ratio; BMI, body mass index; BRI, body roundness index; CI, conicity index; DAI, dysfunctional adipose index; TAA, tissue adiposity area; VAI, visceral adiposity index; WC, waist circumference; AdipoQT, total adiponectin; AdipoQHMW, high-molecular-weight adiponectin.

**Table 2:** Cross-validation results for recursive feature selection.

	Full Set			Reduced Set	
	AUC-ROC	Accuracy	Size	AUC-ROC	Accuracy
LR	0.865 (±0.098)	0.793 (±0.073)	6	0.897 (±0.063)	0.8127 (±0.069)
SVM	0.861 (±0.075)	0.778 (±0.077)	7	0.882 (±0.073)	0.7959 (±0.078)
XGboost	0.881 (±0.058)	0.851 (±0.055)	10	0.882 (±0.063)	0.854 (±0.052)
RF	0.882 (±0.067)	0.864 (±0.045)	7	0.893 (±0.061)	0.875 (±0.041)

The area under the receiver operating characteristic curve (AUC-ROC) and accuracy are presented, along with their respective mean values and standard deviations in parentheses, which were calculated through cross-validation on both the full and reduced sets.

**Table 3:** Evaluation metrics of the models trained.

Metrics	LR	RF	XGBoost	SVM
AUC-ROC	0.910 (±0.048)	0.886 (±0.068)	0.867 (±0.063)	0.881 (±0.068)
Sensitivity	0.816 (±0.069)	0.781 (±0.088)	0.759 (±0.090)	0.798 (±0.162)
Specificity	0.859 (±0.146)	0.827 (±0.155)	0.778 (±0.179)	0.795 (±0.098)

The AUC-ROC, sensitivity, and specificity are presented with their respective mean values and standard deviations in parentheses, calculated through cross-validation.

**Table 4:** Model’s performance in the test set.

Metrics (95%, CI)	LR	RF	XGBoost	SVM
Accuracy	0.86 (0.74–0.94)	0.88 (0.76–0.96)	0.80 (0.67–0.90)	0.83 (0.70–0.92)
Sensitivity (Recall)	0.85 (0.62–0.97)	0.85 (0.62, 0.97)	0.70 (0.46–0.88)	0.75 (0.51–0.91)
Specificity	0.87 (0.71–0.96)	0.91 (0.75, 0.98)	0.88 (0.71, 0.96)	0.88 (0.71, 0.96)
PPV	0.80 (0.58–0.95)	0.85 (0.62, 0.97)	0.78 (0.52, 0.94)	0.79 (0.54–0.94)
NPV	0.90 (0.74, 0.98)	0.91 (0.75, 0.98)	0.82 (0.65, 0.93)	0.85 (0.68–0.95)
Balanced Accuracy	0.86 (0.74, 0.94)	0.88 (0.77, 0.96)	0.81 (0.67, 0.90)	0.83 (0.70–0.92)
AUC-ROC	0.93 (0.86–0.99)	0.94 (0.88–1.00)	0.954 (0.87–1.00)	0.931 (0.86–0.99)

Performance of the four supervised classification algorithm models, LR, logistic regression, RF, random forest, XGBoost, and SVM, support vector machine. Abbreviations: PPV, positive predictive value; NPV, negative predictive value; AUC-ROC, area under the receiver operating characteristic curve; CI, Confidence intervals.

## Data Availability

The data presented in this study are available on request from the corresponding author. The data are not publicly available due to privacy or ethical restrictions.

## Appendix A

**Table A1:** Hyperparameter Settings Used for Training Each Model.

Model	Method (Caret)	Hyperparameter Tuning
Random Forest (RF)	rf	mtry = 2
Support Vector Machine (SVM)	svmLinear	C = 0.01
eXtreme Gradient Boosting (XGBoost)	xgbTree	nrounds = 200, eta = 0.1, max_depth = 3, gamma = 0, subsample = 0.8, colsample_bytree = 0.8, min_child_weight = 1
Logistic regression (LR)	glm (binomial)	-
